# Importance of customer adjustment regions in the non-clinical property of thought: A home examination in low and high-income areas of Mashhad

**Published:** 2015

**Authors:** S Fazaeli, M Yousefi, SH Banikazemi, SAH Ghazizadeh Hashemi, A Khorsand, Sh Badiee

**Affiliations:** * Department of Medical Records and Health Information Technology, School of Paramedical Sciences, Mashhad, University of Medical Sciences, Mashhad, Iran,; ** Department of Health Economics and Management Sciences, School of Health, Mashhad University of Medical Sciences, Mashhad, Iran,; *** Health Strategic Research Center, School of Health, Mashhad University of Medical Sciences, Mashhad, Iran,; **** Department of Complementary and Chines Medicine, School of Persian and Complementary Medicine, Mashhad University of Medical Sciences, Mashhad, Iran

**Keywords:** responsiveness, care quality, consumer adjustment, homes

## Abstract

Responsiveness was proposed via WHO as a fundamental sign to evaluate the enforcement of wellness practices and evaluates with a standard organization of fields that are classified to 2 principal classes “Respect as characters” and “customer adjustment”. The current research included the value of customer adjustment areas in low and high-income communities of Mashhad. In the current descriptive research, an example of 923 families was chosen stochastically of 2 low and high pay areas of Mashhad. WHO survey employed for information gathering. Regular rate reviews and Ordinal Logistic Regression (OLR) applied for information investigation. In overall, respondents chose basic amenities quality as the primary area, and the path to social care networks recognized as the wicked primary area. Families in high-income states obtained higher areas of immediate notations and selection associated with low-income. There is a meaningful correlation among parameters of ages, having a part whom required care and self-imposed health via the ranking of customer adjustment areas. The investigation of the homes’ viewpoint concerning the classification of non-clinical perspectives of care quality, particularly while confronted by restricted sources, can assist in managing enterprises towards topics that are more relevant and results in the development of the wellness policy achievement and fecundity.

## Introduction

WHO recognized responsiveness as one of the principal objects to whatever fitness policies participate in developing the community well-being and in promoting its judgment in a regular process beyond nations, with promoting a standard collection of regions [**1**-**3**], that classified to 2 principal classes [**4**]: “Regard to individual” and costumer adjustment”. Regard to characters attributes to the capturing intent the moral features of the communication among people and the health policy and comprises 3 sub-parts: significance, independence, and confidentiality [**4**,**5**]. Customer adjustment measures the elements of customer entertainment and comprises 4 sub-parts: ready care, primary amenities quality, entrance to social provides systems (over inpatient supervision) and care providers choice [**3**,**6**]. 

WHO declared that these areas have a ‘‘universal’’ point, indicating that they are essential for all individuals, culture regardless, age, sex. WHO declared a grave matter regarding examining the applicants’ preferences concerning various features of health settings [**6**].

Several investigations revealed that normally there were differences in preferences among private cases and within cases from various societies and the well-being care systems, and personal attributes like knowledge, health situation, gender and age [**7**,**8**]. Plus some researches have stated limited connections among advantages and people (or home) and the socio-finance features [**9**-**11**]. This variation may result in disagreements and even absence of pleasure [**6**]. Accordingly, delimiting the relevant value of non-clinical perspectives of care quality amongst multiple sub clusters (according to income, lifestyle, etc.), could be helpful in implementing a conventional analysis of health settings applicants’ requirements and assist in optimizing the health policy sources allocation [**12**-**14**]. Notwithstanding the significance of this point, we have a few types of research in this region related to other wellbeing policy topics [**6**,**11**] and the prior investigations have been done on the idea of surveying cases entertainment [**15**-**17**].

The primary purpose of the current research is to discover the relevant point of the sub-parts relevant to the area of consumer adjustment of non-clinical features of care quality “responsiveness” between chosen regions of Mashhad.

## Methodology

This descriptive and cross-sectional research conducted in 2014. Homes that were native in the high and low-pay area of Mashhad are the demographic people of the research [**18**]. The example dimension for each area determined by Cochran Example Dimension Equation (p=.5 for highest variability, 95% trust straight, and ±5% accuracy). Lastly, the example dimension is of five hundred homes in each area (overall one thousnd homes). 

The multistage sampling employed for example choice. After the resolution of levels, each group divided into groups with alike features (city section), each section being the region surrounded by 4 ways. Next, the researcher designated the number of examples of each group on a routine base between the homes in the selected districts. 

The instrument used in the present work is the WHO survey (consists a responsiveness model containing survey regarding to the “value of the responsiveness zones of the communities opinion” and homes demographic features). The survey tr anslated into Persian and its reliability and validity was confirmed in the research of Rashidan[**11**]. **Table 1** presents expression of the costumer adjustment parts in the survey [**6**].

**Table 1:** Sub-elements Descriptions of customer adjustment

Sub-elements of customer adjustment	Expressing
Quick Notice	• having a fair range and trip time of your place to the well-being care supplier
	• reciving rapid care in urgent
	• small expecting periods for meetings and deliberations, and taking inquiries done immediately
	• small expecting programs for non-emergency operation
Selection	• remaining ready to take your doc or attendant or another character normally giving your well-being watch
	• remaining ready to move to another site for wellness watch if you desire
Quality of Basic Amenities	• having the adequate area, seats and new air in the standing place
	• having a decent equipment (involving decent bathrooms)
	• having wholesome and good food
Social Support	• let to be provided food and other presents via relatives in hospital
	• let to have freedom of religious actions

Qualified participants (at the age of eighteen and upper, optionally parents) were chosen as respondents. Investigators prepared since the origin of information gathering in the current research, regarding the research question, issues, preservation of confidentiality of home’s data, sampling techniques, and questioning approach. Consequently, in the initial meeting, the interviewer provided some data to the partneraccording to the research model (consists a report of the research purposes, sponsor, and issues, etc.). The fulfillment of the investigator caught within 15 - 25 minutes. All partners asked to approve or sign a versed permission application. If a home did not attend to partake in the research or is not being at the place after 3 times applying, according to the example choice guideline, it was renewed by a distinct home. The 5-point Likert rate was implemented. Furthermore, the present research appreciated via the Ethics Council of the Medical Sciences Mashhad University. The normal repetition investigations stated for any consequence issue by area and, the socio-demographically features of homes involved: age, sex, health situation (self-stated wellbeing), training. OLR applied to evaluate the performance of the 10 parameters on the homes’ opinion concerning the value of the customer’s adjustment sub-parts. Total the studies conducted by utilizing SPSS 19.

**Findings**

A sum of 443 homes in high-pay and 480 homes in low-income areas formed the surveys. The analysis of the demographic information of partners determined that there is at least 1 character below the age of twelve in about 40 percent of the homes. Higher than 61 percent of them stated their health situations as great and excellent. The other demographic information exhibited in following **table**. 

**Table 2:** Sub-elements Descriptions of customer adjustment

demographic features of the research example		Quick notice		selection		fundemental amenities quality		social support	
		n%	z (sig.)	n%	z (sig.)	n%	z (sig.)	n%	z (sig.)
zones: high / low pay	low (n=480)	47.6	-0.736 (0.462)	41.4	-2.792 (0.005) **	60.4	-0.872 (0.383)	31.6	-2.741 (0.006) **
	high (n=443)	49.4		50.5		58.5		26.4	
Sex	male (n=448)	48.0	-0.803 (0.422)	47.6	-0.876 (0.381)	56.5	-1.805 (0.071)	29.3	-0.201 (0.841)
	female (n=441)	49.5		44.3		62.6		29.1	
<12 years partner living in the homes	yes (n=383)	47.4	-0.079 (0.937)	45.0	-0.820 (0.412)	60.4	-1.130 (0.258)	29.0	-0.680 (0.497)
	no (n=535)	49.1		46.1		58.9		29.4	
self-assessed care	great and excellent (n=559)	47.6	0.455 (0.797)	45.0	0.171 (0.918)	59.9	0.311 (0.856)	30.9	9.958 (0.007) **
	moderate (n=285)	50.7		46.3		59.2		25.0	
	bad and very bad (n=53)	48.1		53.8		58.0		32.7	
65+ years partner living in the homes	yes (n=262)	50.6	-0.629 (0.529)	48.9	-1.496 (0.135)	56.7	-1.258 (0.208)	28.7	-0.441 (0.659)
	no (n=648)	47.5		44.3		60.5		29.0	
partner via needed care living in the homes	yes (n=252)	58.1	-2.724 (0.006) **	51.4	-1.516 (0.130)	59.4	-0.053 (0.957)	33.6	-1.918 (0.055) *
	no (n=656)	45.3		43.8		59.5		27.7	
using the health provides in the last year/ more than 1 year before	Over the last year (n=716)	50.7	-2.148 (0.032) *	46.8	-1.188 (0.235)	59.7	-0.256 (0.798)	29.8	-2.322 (0.020) *
	more than 1 year prior (n=179)	41.5		42.4		59.9		26.6	
Insurance	have (n=558)	50.5	-0.285 (0.775)	45.0	-2.053 (0.040) *	60.7	-0.103 (0.918)	30.8	-1.317 (0.188)
	do not have (n=289)	49.8		49.8		61.1		25.9	
literacy	0-6 (n=80)	42.9	1.908 (0.385)	35.9	3.887 (0.143)	46.2	8.301 (0.016)*	34.6	19.197 (0.000)**
	6-11 (n=494)	48.3		44.9		60.8		33.2	
	12 < (n=323)	51.9		50.2		63.1		22.4	
**Link is obvious 0.01 stage (2-tailed).									
* Link is obvious 0.05 stage (2-tailed).									

The outcomes showed that partners recognized the quality of primary facilities as the significant sub-part between various customer’s adjustment ones, and after that quick notice, selection, and social provision has the effect sequentially.

By using **Table 1**, we can observe a meaningful relationship among areas, education, and self-assessed well-being via the influence of material assistance (P-Value ≤ 0.01). Besides, there is an analytically meaningful distinction among great rates in material assistance and the application of wellness settings or having a constituent who required care in the home (P-Value ≤ 0.05). 

Furthermore, notable variations observed in arranging a quick notice regarding utilizing wellness settings in the prior year/ more than 1 year before and having a partner who needed watch in the home (P-Value ≤ 0.01). As shown in **Table 1**, the amenities quality is notably linked to the responder's knowledge (P-Value ≤ 0.01). Furthermore, the quality rate of selection of the supplier is mainly distinctive among the 2 areas (P-Value ≤ 0.01) and more among the homes via and without support.

**Table 3** presents that demographic parameters influence the choosing customer adjustment sub-elements as critical.

**Table 3:** Choosing customer adjustment as critical [via 95 percent reliability intervals], from the OLR

Parameters	B	S.E.	Wald	Sig.	95% Confidence Interval	
					L Bound	H Bound
Years old	0.008	0.003	6.231	0.013	0.001	0.017
Self-estimated wellbeing	-0.169	0.076	6.532	0.012	-0.543	-0.043
Training	0.012	0.014	1.209	0.269	-0.012	0.043
More payment	0.00	0.00	1.65	0.221	0.00	0.00
Upper home dimension	-0.029	0.029	1.291	0.243	-0.100	0.030
High pay zone	-0.002	0.226	0.000	1	-0.500	0.478
Women as answering	-0.119	0.110	1.459	0.234	-0.331	0.080
66 > years members being in the home	0.310	0.141	4.429	0.038	0.015	0.454
13 < years members being in the home	0.051	0.131	0.169	0.681	-0.181	0.269
Members who required watch in the home	-0.301	0.119	5.691	0.015	-0.511	-0.049
Pattern Index: LR χ2=23.51 (P. value=0.013), Pseudo R-Square=0.046. (Log-log.)						

These studies revealed that age had a particular impact on the stage of interest of the patient adjustment factors. Self-assessed well-being of participant and being a person who require watch in the home had an inverse impact on the stage of value of the costumer’s orientation sub-parts which were stated via the homes.

## Discussion

Responsiveness represents regard for individual rights in the wellness watch methods and evaluates the attainment stage of certain beliefs of persons from the wellbeing arrangement [**19**].

Responsiveness has 2 central sections, and the present research discovered the relevant value of each factor linked with the customer’s adjustment of the view of the homes in low and high-pay areas. Ordering these fields of the view of characters among various financial, human and cultural features has been featured in numerous investigations [**6**].

Usually, the outcomes in the current research revealed that essential amenities quality was chosen as the outstanding part from the view of partners as well as the investigations carried via Rashidian in area 17 of Tehran, Karami between heart problem cases in the hospital and Kowal in Asia (2011)[**20**]. 

The agreement of these issues does not imply the similar prospects, though these issues determined that the essential amenities quality is the significant part related to other parts of the consumer’s adjustment. But, regarding the measurement scale of the value stage of the sub-parts of customer’s adjustment, the value of these fields cannot be shown in multiple investigations.

The later feature in the present research is the identity of this preference among low and high-pay areas that proved that even homes who live in areas via low-pay, further supposed to get assistance via a proper service quality. It can be given more consideration to understanding that the vital part of the outpatient wellness services in Mashhad are alike among homes with high-pay and those in low-one areas. However, also the preferences set and according to the outcomes from the WHO’s global group studies of “wellbeing process responsiveness” in 42 zones, that was stated in 2008, the distinctive region for Iranian partners is quick notice (32%) [**6**]. This finding can also be observed in some other investigations [**6**,**19**,**21**,**22**].

Conclusions of the current research were compatible via the earlier examinations in Iran and were distinctive from the investigations outside Iran in establishing the advantages.[**6**]. 

The essential amenities quality not only changes the case’s aid, however, is further attached to the response of improving wellbeing, and quickening in the healing manners [**23**]. But, some investigations have demonstrated that there was a break within the cases’ requirements and entrance to primarily wanted facilities also in advanced nations. The undesirability of essential facilities might place the inmate in danger [**24**].

In his research, Valentine explained that perspective preferences in the responsiveness areas in the area of customer adjustment are more correlated with the geographic scope as well as the human growth level and, in some instances, the health expenditure level. Furthermore, in the current research, the vital connection with the kind of place and giving notice to the right selection statistically recognized. It means that characters remaining in the low-pay area have the more advantage for the selection related to the high-pay area [**6**]. 

Some investigations discovered that adult respondents give notice to independence little more than youthful ones. In the current research and the research carried out via Rashidian there is no clear link among demographic characteristics and independence of person [**23**]. In the research on 8 EU zones, Coulter indicated that majority of the people (upper than half) wanted the pattern of adjoining decision-making and 30 percent of them upper 55 age, admitted that the Doc must say [**24**].

In his research, like in any other investigation, there are some restrictions. The weak enthusiasm of homes to partake in these sorts of investigations is one of the chief restraints this research. To surmount these restrictions, we examined to resolve the proper time by a representative from the homes to perform the survey, utilize the promotional means, and increase the interaction experiences of interviewers. Another restriction is the cultural problems over a visit at home that fixed via instructing surveyors and employing in same genders, and getting the needed legal recognition. Furthermore, the deep connection among point is prioritizing and personal properties may be partly defined via the elimination of personal features such as ethnicity that was discovered to be a major determinant in some investigations [**25**].

Management producers in the wellbeing process can utilize these decisions in prioritizing their works when confronted via source limitations [**5**,**26**]. Since out of the advantages understanding in the area, attempting to reform and change the wellbeing process administration that usually concentrates on tangible advantages, like revenues and expenses, might be misguided. It might be because the usual information cannot explain many of the expenses like the loss created by the absence of a timely convenience of cases to required services or created via the absence of sufficient essential amenities quality as an urgent priority in action evaluation. Accordingly, the configuration of suitable mechanisms that enables the prioritizing by characters to plan to do the change, the wellbeing policy is one of the relevant areas of plan planning in development of wellbeing process responsiveness.

**Acknowledgment**

This analysis would not have been conducted without the plentiful participation of the Mashhad residents. We got financial support of Medical Sciences Mashhad University.
